# Expressive writing in a Saudi university English foreign language (EFL) classroom: Evaluating gains in syntactic complexity

**DOI:** 10.12688/f1000research.121577.1

**Published:** 2022-06-30

**Authors:** Shatha Ahmed Abdulaziz Alkhalaf

**Affiliations:** 1 Department of English and Translation, College of Sciences and Arts, Qassim University, Saudi Arabia

**Keywords:** : Expressive writing, EFL, general writing, Saudi learners, syntactic complexity in English.

## Abstract

**Background: **This study determines if the English foreign language (EFL) Saudi students achieve greater syntactic complexity when they engage in expressive writing than when they write about a general topic.

**Methods:** This study employs an *ex post facto* research design to compare the writing output of EFL learners. The sample comprised of 24 college students enrolled in an English writing course, at Department of English and Translation, College of Sciences and Arts, Qassim University, Saudi Arabia for the academic year 2021-2022. The participants were assigned randomly, and their writing was analyzed using the computer software named Web-based L2 Syntactic Complexity Analyzer. Lu’s (2010) four board element of syntactic complexity and 14 units is employed to analyze the data.

**Results:** Results show that students achieve higher syntactic complexity when engaging in writing on emotional topics (expressive writing) than when writing on general topics. Further, analysis shows that students' emotional writings are significant on three syntactic complexity measures, i.e., length of production units; amount of subordination; and phrase sophistication. The fourth measure, i.e., coordination, does not reflect significant differences between their expressive writing and general writing.

**Conclusions:** The study's implications are expected to aid EFL instructors and curriculum designers in successfully implementing language education, particularly in writing, in the Saudi context. In line with the input hypothesis, this research suggests that writing about personal emotional events may enhance the quality of language two (L2) writing by increasing syntactic complexity. In this dimension, this study could be additional evidence of the Krashen hypothesis.

## Introduction

Creative writing skills enhance one’s capacity to retain knowledge, link concepts, and synthesize information in new ways. In academic settings, its importance cannot be understated not only as a language enhancement exercise, but also, in relieving academic stress. Expressive writing is part of the writing curriculum in English foreign language (EFL) courses in Saudi Arabia but its role in enhancing learners’ ability in forming complex syntactic structures in English has so far not been researched.

The input hypothesis of Krashen suggests that acquiring a language is a natural process in which adults and children can subconsciously obtain written or spoken proficiency. Krashen (1985) asserts that language learners should focus on the meaning rather than the form, hence, acquiring a language depends upon meaningful interaction in the target language. Furthermore, as a huge corpus of research has shown, language two (L2) writing quality ( Al-Ahdal and Abduh, 2021; Taguchi *et al.,* 2013) and grammatical complexity ( Lu, 2011) are linked with L2 proficiency studies ( Ortega, 2000, 2003; Wolfe-Quintero *et al.,* 1998). Syntactic complexity may be used to distinguish between levels of skill and to predict the quality of writing in a second language. Syntactic complexity in L2 writing may be impacted by a range of learner, task, and context-related factors such as subject matter (genre), preparation time, and instructional setting among others ( Ellis and Yuan, 2004; Sotillo, 2000; Yang *et al.,* 2015). As a result of these studies, we have gained a better understanding of how L2 writing research and instruction may best utilize the concept of syntactic complexity. In line with the input hypothesis, this research suggests that writing about personal emotional events may enhance L2 wiring by increasing syntactic complexity. Thus, this paper could be additional evidence of the Krashen hypothesis.

Studies on the effect of learners’ first language (L1) and other learner-related factors on the difficulty of L2 writing show that, in order to make informed judgments, it is vital to pay particular attention to any L1-related variations. Until now, few studies have examined these sorts of differences in depth. In most research comparing the complexity of writing by non-native speakers (NNS) and native speakers (NS), learners’ L1 background has not been included as an independent variable; rather, the studies have either looked at a homogenous L1 group or regarded all NNS learners as one group (e.g., Ai and Lu, 2013). Crossley and McNamara’s (2012) pioneering study revealed that there were statistically significant differences in the complexity of L2 English writing by four L1 groups. Syntactic complexity was measured just by the average number of words preceding the principal verb in the phrase. Thus, our current understanding of the grammatical complexity variations in L2 writing that are produced by variances in L1 writing has been severely limited.

### Literature review

There has been a lot of research on how difficult it is to write in a second language, but one thing that has not been looked at very carefully is how difficult it is for people to write in their first language (L1). There is already a lot of research on how people who speak more than one language write in their second language (e.g., Al-Ahdal and Alqasham, 2020; Edelsky, 1982; Lally, 2000; Lefrançois, 2001; Paquot, 2013; Uysal, 2008; van Weijen *et al.,* 2009). Some of the characteristics that have been looked at are as follows: idea generation ( Lally, 2000); information structure ( van Vuuren, 2013); rhetorical patterns ( Uysal, 2008); syntactic structures ( Rankin, 2012) and; lexical bundles ( Paquot, 2013; Rankin, 2012). Further, Lefrançois (2001) said that in addition to knowing how to write and read, the way grammar and syntax work, how general techniques work, and how cultural schemas work in the first language could all have an effect on writing in the second language. With written data from the International Corpus of Learner English (ICLE) Version 2.0, the research in a recent book edited by Jarvis and Crossley (2012) looked at linguistic patterns that are unique and different for people who speak a different language than their first language (ICLE 2.0; Granger *et al.,* 2009). Specifically, they said that patterns of coherence, lexical style, n-grams, mistakes, conceptual understanding, and syntactic complexity, might all be used to identify people who speak a different language than their first language.

A thorough grasp of these distinctions, on the other hand, would have significant consequences for L2 writing research and teaching in general. Research on L1-related differences in L2 writing will benefit from this understanding if it is applied, and researchers will be able to determine whether and how learners’ L1 should be controlled or taken into account when collecting, analyzing, and interpreting data, among other things, in syntactic complexity research. We may now re-examine previous claims about the link between syntactic complexity in L2 writing and L2 competency that were made without considering the potential of an L1 influence. This is a good thing! L2 writing pedagogy can benefit from an appreciation of these differences, which can help teachers see that the same patterns of syntactic complexity aren’t always indicative of L2 proficiency in the same way for learners from varied L1 backgrounds. As a result of this knowledge, they will be able to better address the difficulties associated with syntactic complexity for students from a range of L1 backgrounds. An in-depth look at how changes in L1-related syntactic complexity affect L2 writing will also be helpful in the current study on learner texts’ automatic native language identifiability ( Tetreault *et al.,* 2013).

Emotions also have a crucial effect in the grammatical complexity of second language writing, as several studies have demonstrated. Studying 2,600 Chinese college students’ essays, Wang and Curdt-Christiansen (2016) found that emotions and grammatical complexity in second language writing are linked. They concluded that emotional writing affects EFL students and leads them to write simpler syntactic clauses and sentences.

Writing prompts have been shown to have a significant impact on the emotional and grammatical complexity of second language writing. Tabari and Wang (2022) recruited 53 advanced-mid ESL students by inviting them to write essays on the COVID-19 pandemic and non-pandemic topics alike. The second language writers’ emotionality and vocabulary complexity were found to be affected by writing prompts. The study also shows that emotions play an important role in second language writing which may be taken into consideration in teaching and learning a second language.

### Research gap

Since there no studies that examine Saudi EFL college students’ performance in expressive writing as a factor in their ability to compose complex syntactical structures in English, the current study aims to fill this gap by examining L2 writing of EFL Saudi students in order to discover if there is a substantial link between writing on emotional topics and syntactic complexity. The length of the production unit, the degree of subordination, the degree of coordination, the degree of phrasal sophistication, and the overall complexity of the phrase are all employed by the researcher to gauge syntactic complexity (see Table 1).

**Table 1.** Lu’s (2010) 14 indices for language two (L2) syntactic complexity with codes.

Dimension	Measure/Index	Code
Length of production unit	Mean length of clause Mean length of sentence Mean length of T-unit	MLC MLS MLT
Amount of subordination	Clauses per T-unit Complex T-unit per T-unit Dependent clauses per clause Dependent clauses per T-unit	C/T CT/T DC/C DC/T
Amount of coordination	Coordinate phrases per clause Coordinate phrases per T-unit Verb phrases per T-unit	CP/C CP/T T/S
Degree of phrasal sophistication	Complex nominals per clause Complex nominals per T-unit Verb phrases per T-unit	CN/C CN/T VP/T
Overall sentence complexity	Clauses per sentence	C/S

### Research question

Thus, the hypothesis of this study proposes that the emotionality of the writing topics positively affect the writers’ productions by increasing the syntactic complexity in L2 writing overall. It can be hypothesized that, ‘Saudi EFL learners achieve better syntactic complexity on emotional subject that other general subject’.

Consequently, the current study attempted to answer the following research question:

• Do EFL Saudi students achieve better syntactic complexity when they engage in expressive writing (write about an emotional subject) than when they write about a general topic?

## Methods

### Ethical statement

Ethical approval for this study was given by the Scientific and Ethical Committee of the Department of English and Translation, College of Sciences and Arts, Qassim University, Saudi Arabia. Verbal informed consent was obtained from all participants prior to participation in the study to the effect that their data will be published. The reason behind getting only verbal consent from the participants as notified in the consent letter is for the global interest of research.

### Research design

This study employed an *ex post facto* research design. *Ex post facto* research design is also referred to as correlational research ( Griffee, 2012). On the other hand, Tavakoli (2012) stated that in *ex post facto* research, the researcher sets hypotheses as recommendations at the end of the study. Furthermore, Cohen *et al.* (2007) affirmed that *ex post facto* is used instead of experimental research whenever it is impossible to control variables. In this study, a two-paragraph essay was required from the participants to be produced, one of which discussed their feelings on keeping a pet and the other of which told the tale of an emotionally charged incident that had occurred in their lives. Using Web-based L2 Syntactic Complexity Analyzer, 2010, a comparison was done between those two paragraphs in order to identify in which case syntactic complexity was higher.

### Participants

The exercise was completed by all the 50 students taking the English writing course, and 33 were randomly taken as sample participants. However, when the data were analyzed it was found that nine of these samples were not viable as they were either incomplete or fell short of the requisite length. Hence, these were removed. The respondents for this survey were all females selected through the use of a random sampling procedure because in Saudi Arabia, females study in segregated classrooms, with no male students around. The class for this study was a female only class due to this. The randomness was achieved using an application called Raosoft ( Raosoft, 2004), in which all the 50 students wrote the two paragraph essays and only the written essays of 24 of them were chosen for analysis. It was actually very difficult to include all the 50 students written paragraphs for analysis. The participants were all studying at the second language level (L2) and they shared the same or comparable cultural backgrounds and ability level in English. They were all between the ages of 19-21 studying in their third year, semester six, which is referred to as level six in Saudi Arabia. Before participation, the students were explained the aims of the research and their consent to participate in the study was sought. The rationale behind using random sampling was that this way everyone in the population was guaranteed a fair chance at being selected. The technique is simple to follow and is considered fair because any individual can be chosen ( Berndt, 2020). It was determined using Raosoft that the respondents’ sampling representation would be the most accurate.

### Data collection

Amongst free writing tasks, short compositions are most favored in Saudi EFL classrooms given the fact that they can be finished in one sitting by the students without break in the train of thoughts and are also more feasible for the teachers to provide early and detailed feedback on. For the purpose of this study, the students were asked to submit a short composition in two paragraphs at the start of the summer semester on 15 June 2021 as part of the writing class, and the second in the following week, on 22 June. In the first paragraph, they were asked to write on their experience to have a pet at home and in the second, they were instructed to narrate a story/situation that they experienced in life. All the students in this course, studying in their third year of the English department, wrote the two required paragraphs in the first class of the semester, but only the responses of 24 of them were added to the Web-based L2 Syntactic Complexity Analyzer (L2SCA) after random selection, as shown in Table 1. Further information about the data processing was included in the analysis section. The setting for data collection was Qassim University and the activity was in-class. Thus, in all, there were 24 emotional and an equal number of narrative manuscripts. All data were initially in the form of paper submissions, these were later converted to word documents in the computer by the researcher herself to rule out the possibility of inadvertent error corrections or any other changes in the original manuscripts. These were then analyzed as discussed in the following sections.

### Data analysis

A computer tool called the L2SCA by Lu (2010), which is built to assess English writing samples for syntactic complexity using the 14 metrics ( Table 1), was utilized to analyze all of the datasets in this study. L2SCA was seen as the most suitable software due to its free availability, its ability to process files in batches, and its high degree of dependability when it comes to processing files.

There are 14 syntactic difficulty indices that are derived based on the frequency counts supplied by L2SCA for each writing sample in this study. According to the frequency counts, these are: verb phrases, sentences (which include dependent clauses), tenses (T units), complex tenses, coordinate phrases (complex nomen), and complex nominatives. L2SCA’s correlations with human annotators’ syntactic difficulty scores ranged from .834 to 1.000, while the accuracy of structural unit recognition varied from .830 to 1.000. The level of significance that has been determined is 0.05.

## Results

There was a total of 33 participants randomly selected, however, only the paragraphs of 24 participants were analyzed in this study as nine were removed from the analysis due to being incomplete or not to the required level. Participants were between the ages of 19-21 studying at level 6 at Qassim University in the female only English writing course. As two essays were submitted from each student, the total number of essays included in this study was 48 ( Alkhalaf, 2022b).

### Differences between writings on emotional and general topic

In order to compare the level of syntactic complexity, we looked at both emotional and broad topics. All 14 indices of syntactic complexity were shown to be statistically significant by the t-test. Syntactic complexity may be broken down into five distinct categories: length, subordination, coordination, and phrasal sophistication. These variations (or sentence structures) are analyzed in the next section.

*Length of production unit*

For the three-length metrics, as shown in Table 2, the emotional and wide themes had considerably different means and standard deviations than the narrow and emotional topics. When it comes to MLS, in the emotional topic students achieved a general mean score of 22.309 and standard deviation of 5.584 whereas in the general subject they scored a mean of 14.672, and standard deviations 3.574. As can be seen, there is a range difference between students’ emotional and general writing, at 7.637. Furthermore, there is a significance level of.001 between the two types of writing. When it comes to MLC, students scored a mean of 10.624 in the emotional topic and standard deviation of 1.217. The mean score and standard deviation were 8.307,1.984, respectively.

**Table 2.** Length of production unit for the emotional and general topic essays.

MLS				MLC				MLT			
Topic	Mean	SD	p	Topic	Mean	SD	p	Topic	Mean	SD	p
Emotional	22.309	5.584	.001	Emotional	10.624	1.217	.001	Emotional	18.982	4.612	.001
General	14.672	3.574		General	8.307	1.984		General	13.115	2.574	
Range	7.637			Range	2.317			Range	5.867		

The difference between the two scores is 2.317, which seems less than the difference in the MLS. However, the difference is not huge, it still significant as the *p* value showed a significance level of .001.

The third area checked was students’ production in MLT, in the emotional topic, the students got a mean score and standard deviation of 18.982 and 4.612, respectively; they scored in the general subject a mean score of 13.115, and standard deviation of 2.574. The range difference between students’ scores in the two types of writing is 5.867 which is considered as significant as the *p* value is .001. This means that the values for the two topics have significant differences. These results are summarized in Table 2 below.

*Amount of subordination*

Table 3, shows the amount of subordination in students’ writing. The emotional and general themes had significantly different means and standard deviations for all the four subordination assessments when compared to the emotional and broad subjects. The mean for the emotional subject for C/T is 2.108, whereas that for the general topic is 1.583, with a *p* value of .001 for both. When it comes to CT/T, the mean value for the emotional topic is .576 and that for the general subject is .436. The *p* value for both of these measures is .0001. In the DC/C sample, the mean for the emotional topic is .456 and that for the general subject is .345; both have *p* values less than .0001. With a *p* value of .001, the mean for the emotional topic is 1.019 and that for the general subject is .567 for DC/T, respectively. This indicates that there are considerable discrepancies in the values for the two themes.

**Table 3.** Amount of subordination for the emotional and general topic essays.

C/T				CT/T				DC/C				DC/T			
Topic	Mean	SD	p	Topic	Mean	SD	p	Topic	Mean	SD	p	Topic	Mean	SD	p
Emotional	2.108	.685	.001	Emotional	.576	.168	.001	Emotional	.456	.110	.001	Emotional	1.019	.599	.001
General	1.583	.238		General	.436	.127		General	.345	.078		General	.567	.192	
Range	0.525			Range	0.14			Range	0.111			Range	0.452		

*Amount of coordination*

As demonstrated in Table 4, the emotional and wide themes did not have significantly different means and standard deviations from the narrow and emotional topics when it came to the three coordination measures. For CP/C, the mean for the emotional topic is .301, and that for the general subject is .168; both of these mean values are statistically significant at a threshold of .121. With regard to the correlation coefficient (CP/T), the mean for the emotional topic is .528 and that for the general subject is .263, with a significance level of .064 for both. When it comes to T/S, the mean for the emotional topic is 1.186, and that for the general subject is 1.092, with a significance level of .729. This indicates that the numbers for the two topics do not differ statistically significantly.

**Table 4.** Amount of coordination for the emotional and general topic essays.

CP/C				CP/T				T/S			
Topic	Mean	SD	p	Topic	Mean	SD	p	Topic	Mean	SD	p
Emotional	.301	.142	.121	Emotional	.528	.249	.064	Emotional	1.186	.156	.729
General	.168	.087		General	.263	.139		General	1.092	.095	
Range	0.133			Range	0.265			Range		0.094	

*Phrasal and overall sentence complexity*

Table 5 shows that the averages and standard deviations for the four subordination evaluations differ significantly between emotional and wide-base themes. The mean for the emotional subject for CN/C is 1.281, while that for the general subject is .913, with both having a *p* value of .001. The mean for the emotional topic for CN/T is 2.140, whereas that for the general subject is 1.499. Both of these measurements have a *p* value of .001. The mean for the emotional topic in the VP/T sample is 2.862, whereas that for the general subject is 2.075; both have *p* values less than .001. For C/S, the mean for the emotional topic is 2.496 and that for the general subject is 1.748, both with a *p* value of .001. This demonstrates that there are significant disparities in the values assigned to the two topics.

**Table 5.** Phrasal and overall sentence complexity for the emotional and general topic essays.

CN/C				CN/T				VP/T				C/S			
Topic	Mean	SD	p	Topic	Mean	SD	p	Topic	Mean	SD	p	Topic	Mean	SD	p
Emotional	1.281	.357	.001	Emotional	2.140	.709	.001	Emotional	2.862	.934	.001	Emotional	2.496	1.124	.001
General	.913	.272		General	1.499	.477		General	2.075	.396		General	1.748	.301	
Range	0.368			Range	0.641			Range	0.787			Range	0.748		

## Discussion

The current study compared the level of syntactic complexity between students’ writing on emotional and general topics. The comparisons in students’ writing were made in five sub elements. The study found that students achieved higher scores in the emotional essays than they achieved in general topic, however, the differences were significant in only four elements out of five. Students’ expressive writings were more developed than their writings on general topics with reference to length of production unit, amount of coordination, and phrase complexity. Nevertheless, the differences in students’ writing regarding amount of coordination were not significant. These findings indicate that interest and motivation in topics chosen for writing urge students to write well when compared with writing on topics they do not prefer. The findings of this study are in line to some extent with Lu and Ai (2013). According to their findings, when the L1 backgrounds of the EFL learners were taken into consideration, just three of the 14 measures indicated statistically significant variations between the groups. Nonetheless, when learners were divided into groups according to their L1 backgrounds, statistically significant differences in all 14 measures were discovered, and the other groups had significantly different patterns from the other group. L2 writing and L2 proficiency have been explored in a number of research ( Ai and Lu, 2013; Norrby and Håkansson, 2007; Stockwell and Harirington, 2003; Wolfe-Quintero *et al.,* 1998). It is feasible to consistently utilize some measures of syntactic complexity to distinguish levels of L2 proficiency and some to predict the quality of L2 writing, according to these studies. There are newer studies that show L2 writing difficulties can be impacted by several factors, including learner and task-related ones like the subject matter of the assignment, preparation time and instructional context. Writing in L2 requires a highly evolved understanding of grammatical complexity, and the analysis in this study corroborates this.

Several studies, like Badenhorst (2018), which used the reflections of 19 Masters and PhD students engaged in a course called graduate research writing to represent student experiences across a semester, showed results similar to this study. Emotional awareness and decision making were aided by the use of instructional tactics that encouraged students to take control of their own emotional responses to critical feedback. Emotional or expressive writing encourages students to be more creative, innovative, and imaginative.

Additionally, student emotions were managed differently in the Driscoll and Powell’s (2016) study, with some students approaching their learning less emotionally (rational interpreters), others more emotionally (emotional interpreters), and a final group employing metacognitive practices to manage their emotions (metacognitive interpreters or emotional managers). Students’ ability to navigate the complicated emotional terrain of writing in higher education appears to be facilitated by metacognitive ideas of monitoring and control, according to the findings.

### Summary of findings

This study generally determined that the EFL Saudi students achieve better syntactic complexity when they write about an emotional subject than when they write about a general topic. According to all three indicators, the duration of the production unit has statistically significant differences between the two variables. All four variables point to statistically significant differences in the levels of subordination across the various categories. There were no statistically significant variations in the level of coordination measured by the three metrics. Finally, it was discovered that there are statistically significant variations in phrasal and total phrase complexity for all of the measures. The study’s implications will aid EFL instructors and curriculum implementers in successfully implementing language education, particularly in writing, in the Saudi context, according to the findings.

### Recommendations

Emotional intelligence and the capacity to express one’s feelings in writing go hand in hand, according to Castillo *et al.,* (2019). This study lends credence to prior studies that shown how expressive writing may promote positive emotional well-being and how an expressive writing program can improve emotional processing abilities, both of which are important components of emotional intelligence. As a result, the study recommends that expressive or emotional writing be pursued more rigorously in EFL classrooms to enhance the ability of the students to compose syntactically complex structures that will bring their writing closer to that of native users of the language. Additionally, this research recommends to teachers to give more space to expressive writing as writing about one’s feelings might help their students better regulate their own emotions. Individuals appear to be affected in a similar way by writing, whether it is about joyful or sad events. The study also recommends further research that links with past research to help us understand how expressive writing is related to emotional intelligence, which has been established in earlier studies as well.

### Limitations

The study faced the limitation of being focused on an exclusively female sample given the practices prevalent in the Saudi society. The other limitation was the small group of participants. Both of these factors somewhat limit the applicability of the findings of this study.

## Conclusion

The study compared Saudi EFL students’ writing of narrative/general and opinion/emotional essays. It used five elements of analyses and found that students scored higher in the latter category than they scored in the former type of essays, in respect to their use of syntactic complexity. Out of the five sub-elements of syntactic complexity, the differences between students’ writing were found in three elements, i.e., length of production unit, amount of coordination, and phrase complexity. Nevertheless, the students’ use to the amount of coordination was compared in their emotional and general writings; no significant difference was found, though. It is thus recommended that English instructors and curriculum designers make use of topics which are of interest to the students and are part of their life experiences to enhance their writing abilities—topics that are of relevance and significance to them.

## Data availability

### Underlying data

Figshare. Expressive Writing in a Saudi university EFL classroom: Evaluating gains in syntactic complexity. https://doi.org/10.6084/m9.figshare.19632732.v1 ( Alkhalaf, 2022a).

This project contains the following underlying data:

• Shatha –Underlying Data.pdf (Data analysis of narrative and opinion paragraphs).

Figshare. Expressive Writing in a Saudi university EFL classroom: Evaluating gains in syntactic complexity. https://doi.org/10.6084/m9.figshare.20041250.v1 ( Alkhalaf, 2022b).

This project contains the following underlying data:

• Narrative details.rar. (File with the 24 anonymized narrative paragraphs used in this study).
• Opinion paragraphs.rar. (File with the 24 anonymized opinion paragraphs used in this study).

Data are available under the terms of the Creative Commons Attribution 4.0 International license (CC-BY 4.0).
