# Supplementary material for: Expressive writing in a Saudi university English foreign language (EFL) classroom: Evaluating gains in syntactic complexity
Source: F1000Res. 2022 Jun 30;11:723. [Version 1] doi: 10.12688/f1000research.121577.1 (PMC10050904; doi:10.12688/f1000research.121577.1)
Supplement: Peer review history

# Peer review: Reviewer response for version 1

**DOI:** 10.5256/f1000research.133453.r150844

**Published:** 2023-03-28

**Authors:** Jamal Kaid Mohammed Ali

**Affiliations:** 1 Department of English, College of Arts, University of Bisha, Bisha, Saudi Arabia

The paper compares Saudi EFL students' syntactic complexity in writing production due to their contribution in two writing types: expressive writing and writing on general topics. The study revealed that students' syntactic complexity in expressive wring was ameliorated significantly in three of the four tested areas, i.e., length of production units; the amount of subordination; and phrase sophistication. On the contrary, the fourth area, i.e., coordination, had no significant difference between students' production in expressive and general topic writing. 

 The article is well-written and has a good structure. It is well-planned and executed. It demonstrates the field's originality. The goal and results of the current research have been briefly and succinctly summarized in the conclusion; suggestions and implications have been presented at the end of the paper. This manuscript is written in an engaging manner. It addressed one of the most significant ideas. Elements of the articles are well presented and written academically. However, there are some minor suggestions for the improvement of the paper:

1. The abstract seems intriguing. It does an excellent job at establishing the topic and transitioning from one part to the next. However, it is advised to consistently combine the single and plural forms of key phrases (expressive writing, emotional writing, and writing on general topics). It is probably not advisable to use possessive pronoun as used in "study's implications", instead preferably use "Implication of the study".
2. The issue has been introduced in the introduction, which also demonstrates the gap and the significance of this study. In the introduction, there are a few minor errors that need fixing:
  - There are "run on" sentences:
  "its importance cannot be understated not only as a language.... Expressive writing is part of the writing curriculum in English foreign language (EFL) courses in Saudi Arabia, but its role in enhancing learners' ability to form complex syntactic structures in English has so far not been researched."
  The better version would be, "its value as a language cannot be overstated," "In Saudi Arabia, expressive writing is a component of the writing curriculum for English as a foreign language (EFL) classes, although its impact in boosting students' capacity to build complex syntactic structures in English has not yet been investigated."
3. The methodology section is adequate and provides detailed information on the steps taken in the procedure.
4. The results are shown and discussed. The research question is well-addressed. It is recommended that recent references are used to support the findings. It is recommended that the author discuss the previous research results with the current findings.
5. Several pieces of research are discussed in the literature review. They contain important references and some recent studies. However, the second paragraph needs more citations, if possible. It is also recommended that more recent citations should be used. The Research questions can also be shifted into the introduction section. Furthermore, Table 1 should be moved from the literature review to the methods section (data analysis).

Is the work clearly and accurately presented and does it cite the current literature?

Partly

If applicable, is the statistical analysis and its interpretation appropriate?

Yes

Are all the source data underlying the results available to ensure full reproducibility?

Yes

Is the study design appropriate and is the work technically sound?

Yes

Are the conclusions drawn adequately supported by the results?

Yes

Are sufficient details of methods and analysis provided to allow replication by others?

Yes

Reviewer Expertise:

Motivation, Online Learning, Language Skills

I confirm that I have read this submission and believe that I have an appropriate level of expertise to confirm that it is of an acceptable scientific standard.

# Peer review: Reviewer response for version 1

**DOI:** 10.5256/f1000research.133453.r144438

**Published:** 2022-08-01

**Authors:** Mohammed Shormani

**Affiliations:** 1 Department of English Studies, University of Cyprus, Nicosia, Cyprus; 2 Department of English Studies, University of Ibb, Ibb, Yemen

**Evaluation report**

 This research article tackles an interesting aspect of EFL students' writing skills. It highlights the connection between syntactic complexity and the sort of topic students write about, and how this affects students’ L2 syntactic achievement. It is also a valuable addition to the existing knowledge on this particular aspect, and in the field in general.

 Pedagogically, the study provides syllabus makers and curriculum designers with important findings to be considered during the syllabus designing of Writing Courses.

**Some observations to be paid attention to:**

- the repetition of abbreviations.
  please state the phrase and its abbreviation only once: you keep repeating Second language (L2) and First language (L1). Some of these reparations include:
  “…..it is for people to write in their first language (L1).
  Table (1) 14 indices for language two (L2) syntactic complexity
- the abbreviation should be given in the first use:
  Example: “Web-based L2 Syntactic Complexity Analyzer (L2SCA)”.. you gave the abbreviation in the second use.
-  it is better to write the full forms of “MLS”, “MLC”, “MLT” (in the text not in the tables) vis-à-vis their abbreviations/acronyms.
- "These results are summarized in Table 2 below" **replace the word “below” with “above”.**

**Language mistakes:**

 The article should be carefully revised for language mistakes. Some examples of these mistakes are provided as follows (including the section):

- **Abstract**
  comprised of 24 college students enrolled **delete the preposition “of”**
- **Research gap**
  Since there no studies that examine **insert the verb “are”**
- ** Research question**
  “‘Saudi EFL learners achieve better syntactic complexity on emotional subject that other general subject” **replace the word “that” with “than”**
- **Conclusion **
  “the students’ use to the amount of coordination” **replace the word “to” with “of”**
- **Recommendations**
  “…to prior studies that shown how expressive writing” **insert the Aux. “have”**

The conclusion should be a little bit elaborated, stating the five elements of analysis examined … “It used five elements of analyses”, please write these elements in the conclusion for ease of reference.

Is the work clearly and accurately presented and does it cite the current literature?

Yes

If applicable, is the statistical analysis and its interpretation appropriate?

Yes

Are all the source data underlying the results available to ensure full reproducibility?

Yes

Is the study design appropriate and is the work technically sound?

Yes

Are the conclusions drawn adequately supported by the results?

Partly

Are sufficient details of methods and analysis provided to allow replication by others?

Yes

Reviewer Expertise:

My research interests include language acquisition, syntax, phonology, morphology, e-learning, psycholinguistics, etc.

I confirm that I have read this submission and believe that I have an appropriate level of expertise to confirm that it is of an acceptable scientific standard.
